# The first report of two monogenean gill parasites assigned to ‎*Diclidophora merlangi *(Diclidophoridae) and *Loxuroides pricei* ‎‎(Axinidae) from brushtooth lizardfish and red porgy seabream of ‎the Red Sea, Egypt

**DOI:** 10.30466/VRF.2018.30829

**Published:** 2018-06-15

**Authors:** Kareem Morsy, Mohammed Shazly, Mahrashan Abdel-Gawad, Nahed Saed

**Affiliations:** 1 *Department of Biology, College of Science, King Khalid University, Abha, Saudi Arabia; *; 2 *Department of Zoology, Faculty of Science, Cairo University, Giza, Egypt*

**Keywords:** Axinidae, Diclidophoridae, Marine fish, Monogenea, Red Sea

## Abstract

Monogenea is one of the most species-rich groups of parasitic flatworms worldwide with many species described from African freshwater fish. Little is known about the diversity and geographic distribution of monogenean parasites infesting the Red Sea fishes in Egypt. In the present study, a total of 45 specimens of the brushtooth lizardfish *Saurida undosquamis* (family: Synodontidae) and 35 specimens of the red porgy seabream *Pagrus pagrus* (family: Sparidae) was examined for monogenean infestation. Samples were collected from water locations at Hurghada coasts along the Red Sea in Egypt. Two different species were recovered. The first recorded parasite was *Diclidophora merlangi* infesting the lizardfish. This parasite was morphologically similar to the original description for the general body shape, size, shape and arrangement of the clamps and reproduction organs and the number of spines in the lateral groups of the genital atrium, but is distinguished in the host fish which is of a different genus. The second species was *Loxuroides pricei*. The morphological and quantitative data of the isolated specimens and the potential reproductive consequences supported their assignment to *L. pricei* than to the other congeneric species. This parasite can be separated from the morphologically similar *L. sasikala* through having a shorter distance from the anterior extremity to genital atrium or vaginal region, fewer testes and a slightly greater number of spines on cirrus and genital atrium. The two species represented new host and locality records from the Red Sea in Egypt.

## Introduction

Monogenea are small parasitic flatworms mainly found on skin or gills of fish, species infecting marine fish are generally larger than those found on freshwater hosts. The Red Sea is one of the global hotspots for biodiversity, it houses a very high endemism rate compared to adjacent marine regions.^1^ Little is known about the diversity and geographic distributions of monogenean parasites infesting Red Sea fishes in Egypt and there is no information available regarding the type species of these parasites in Synodontidae and Sparidae hosts. The red porgy *Pagrus pagrus*^2^ and* Saurida undosquamis* Richardsn also known as the brushtooth lizardfish are demersal species living mainly in the shallow waters of the Red Sea region. It has also invaded the Mediterranean, being examples of Lessepsian migrants. These fish are of great economic importance in both the Mediterranean Sea and the Atlantic Ocean.^3^^,^^4^ Due to its wide geographical distribution, high market demand and good growth rates, there is a strong interest in breeding this species commercially.^5^^-^^9^ Thus, it is considered as a new candidate species for the diversification efforts of the Mediterranean aquaculture. Among Polyopisthocotylea, families Diclidophoridae Cerfontaine, 1895 (Syn. Diclidophoridae)^10^ and Axinidae^11^ are two of the most predominant families of deep-sea monogeneans.^12^^,^^13^ The detailed history of the family Diclidophoridae was provided^14^ with the type genus, *Diclidophora*^15^ (syn. *Dactylocotyle*)^16^ which was restored, after it had been suppressed for many years in the literature.^17^ Subsequently, separated the type genus, *Diclidophora*^18^ (syn. *Diclidophora*)^15^ into two families, the Diclidophoridae and Dactylocotylidae. Characteristically, members of the genus *Diclidophora *parasitize the gills of fish belonging to three families of Gadiformes including Gadidae, Macrouridae and Moridae and have a complete or well-developed lamellate extension in their haptoral clamps.^19^ There are presently 28 nominal species assigned to the genus *Diclidophora*^18^ (syn. *Diclidophora*).^15^ All other species were transferred to other genera and/or placed in synonymy. The Axinidae^11^ was first recognized at the family level^20^^,^^21^ and divided into 14 genera including *Loxura*,^20^ *Allomonaxine*,^22^ *Axine*,^23^ *Axinoides*,^24^ *Loxuroides*,^25^ etc. Following, the system in which the families Axinidae and Heteraxinidae belong to the suborder Microcotylinea was used.^26^ 13 additional genera have been added to the family Axinidae since Yamaguti’s *Systema helminthum *^17^ was published. Many authors also contributed to the systematics of this family.^27^^-^^34^

 In this paper the haptoral clamps and general morphology of two different species of monogeneans collected from Synodontidae and Sparidae hosts of the Red Sea in Egypt were described. 

## Materials and Methods

During a recent survey of helminth parasites infecting marine fish captured from water locations at Hurghada coasts (Latitude 27° N and Longitude 33° E, Red Sea, Egypt), 45 specimens from the lizardfish (24-30 cm, 100-130 g), *Saurida undosquamis* (family: Synodontidae) and 35 specimens of the seabream (17-26 cm, 75-123 g), *Pagrus pagrus* (family: Sparidae) were examined for monogenean parasites infestation between October 2016-March 2017. To prevent the loss of mobile and temporary ectoparasites, fish were kept alive in aquaria filled with the same water source and examined within few hours. After removing opercula and exposing gill arches, each gill was removed carefully from the fish and immersed in normal saline to remove any excess gill mucus. Monogenean parasites were collected with a Pasteur pipette using a dissecting binocular microscope and kept in 4% formalin till examination. Acetic acid alum carmine was used for staining as described previously.^35^ Drawings were made with the help of a Zeiss microscope supplied with a phase contrast unit.^36^ Helminth identification was confirmed by mounting specimens on slides in drops of ammonium picrate glycerin under coverslips and examining hard parts using light microscopy.^37^ Prevalence and morphometric measurements followed the guidelines described previously.^38^

## Results

**Fig. 1:**
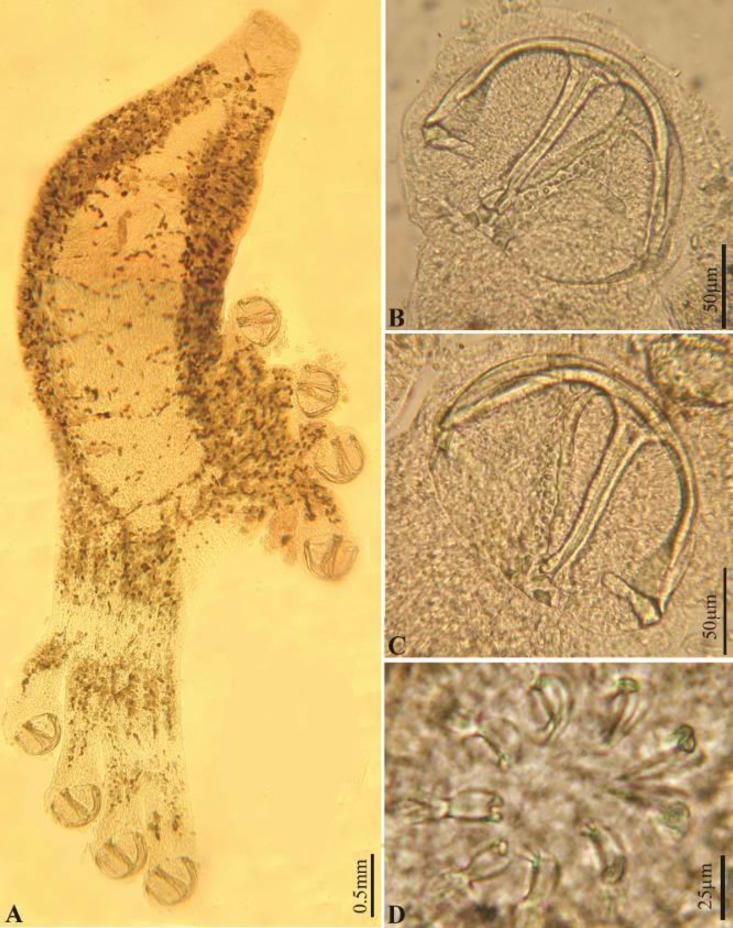
Photomicrographs of *Diclidophora merlangi. *(A) Whole mount; (B) Clamp, dorsal view; (C) Clamp, ventral view; (D) Cirrus armature

**Fig. 2:**
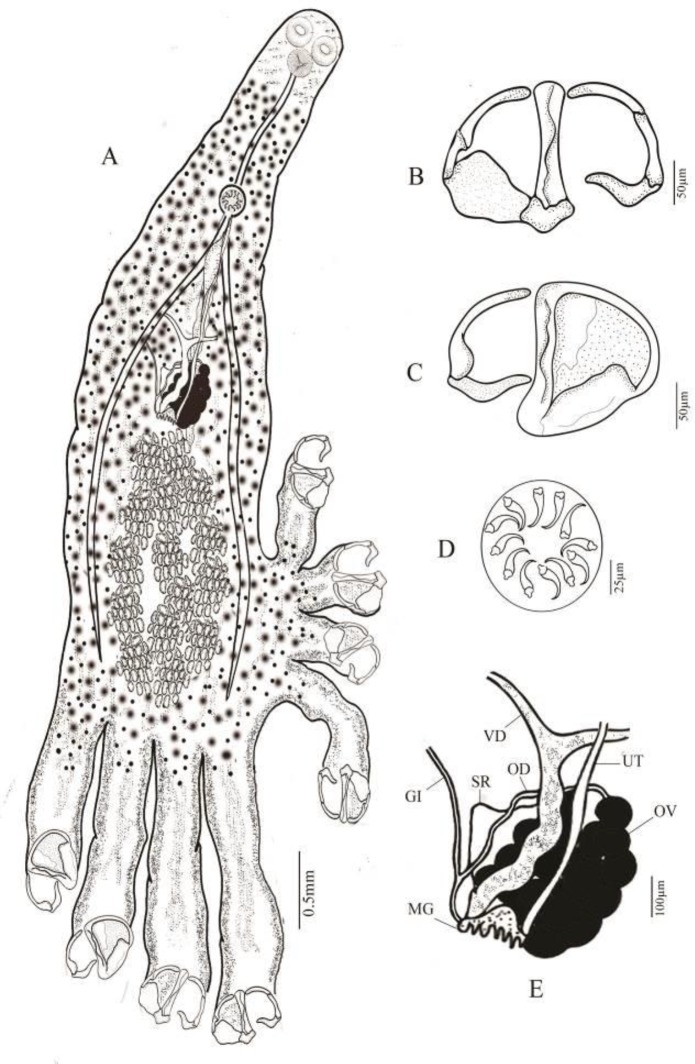
*Diclidophora merlangi* from lizardfish *Saurida undosquamis*. (A) Total view; (B) Clamp, isolated posterior jaw, dorsal view; (C) Clamp, isolated anterior jaw; (D) Detail of female reproductive system: OV, ovary; OD, oviduct; MG, mehlis gland; VD, vitelline duct; UT, uterus; GI, genito-intestinal canal; SR, seminal receptacle; (E) Cirrus armature

The morphometric and anatomical characteristics of *Diclidophora merlangi* are presented in Table 1 and Figures 1 and 2.

Morphological description

Family Diclidophoridae Cerfontaine, 1895

Genus *Diclidophora*^18^

*Diclidophora merlangi* (Kuhn, in Nordmann 1832)^18^

Identification (based on 13 specimens): Body was flask-shaped with distinct broad posterior and elongated, bottle-necked shape anterior end measured 6.00 ±‎ 3.00 (4.00-10.00) mm in diameter. Mouth was small, sub terminal and ventral, provided with two spherical buccal suckers, aseptate. Pharynx was well developed with diameter of 120.00 ± 6.00 (80.00 - 150.00) µm. Esophagus was short. Haptor was not set off from the body proper, bearing four pairs of clamps of unequal size on short peduncles and a terminal lappet. Anterior clamps were largest measured 390.00 ± 10.00 (280.00 - 470.00) and posterior clamps were smallest measured 198.00 ± 10.00 (160.00 - 210.00) µm. Intestinal bifurcation was just anterior to the genital pore. Main caecal branches variably extended into haptoral region, not fused or confluent posteriorly with a length nearly equal. Testes were sub-spherical or irregular, 220.00 ± 40.00(190 - 250) in numbers, in post ovarian median field and entered haptor region to level of first or second pairs of clamps. Vas deferens passed anteriorly in midline to enter the muscular cirrus armed with 15-19 recurved hooks. Ovary was N- or U-shaped, median, in posterior one-half of the body measured 600.00 ± 10.00 (40.00 - 800.00) µm. Seminal receptacle and genito-intestinal canal were on right side of ovary. Mehlis gland was conspicuous, immediately posterior to ovary. Vitelline follicles were small and numerous, coextensive with caeca, not confluent dorsal to testes; transverse vitelline ducts were joined in midline just anterior to ovary to form vitelline reservoir. Uterus was ascended anteriorly in midline, dorsal to vitelline reservoir, terminated at genital pore. Vagina was absent. Copulatory organ consisted of muscular penis with crown of 15-19 much closed grooved and recurved hooks.

Taxonomic summary:

*Type host: *The brushtooth lizardfish, *Saurida undosquamis* (family: Synodontidae).

*Infection Site:* Gill filaments.

*Type locality: *Hurghada coasts along the Red Sea, Egypt. 

*Prevalence:* 18 out of 45 (40.00%) samples of the examined fish were naturally infected.

*Specimens deposited: *Permanent slides were kept in Zoology Department Museum, Faculty of Science, Cairo University, Cairo, Egypt.

*Etymology:* The specific name is derived from *Merlangius merlangus,* the name of the host fish from which the parasite isolated for the first time.

**Remarks. **The specimens described from the lizardfish *Saurida undosquamis* were assigned to the genus *Diclidophora* ^18^ according to the key published previously^39^ where members of this genus are characterized by a posterior haptor not set off from the body proper and a triangular body tapering to the maximum width at the level of the first pair of clamps. Within this genus many species may be compared to the present described. These species are* D. macruri*,^40^ *D. coelorhynchi*,^41^ *D. paracoelorhynchi*,^42^* D. phycidis*, ^3^ *D. luscae*,^16^ *D. esmarkii *(Scott 1901) and *D. merlangi* (Kuhn, in Nordmann, 1832). *Diclidophora macruri*,^44^ a species found on the gills of *Coryphaenoides rupestris* Gunnerus, 1765, differs in having clamps distinctly longer than wide. Morphological differences of *D. coelorhynchi* include its 18 cirrus hooks and pedunculated clamps in which the diagram is not quite united with the lateral sclerites or the base of the central sclerite, so that no ring is formed to support the sucker. The general morphology and clamp structure of *D. paracoelorhynchi* are closest to *D. merlangi*. The *D. paracoelorhynchi's* clamp structure is virtually identical in form to that of *D. merlangi*, except that *D. paracoelorhynchi* has a much larger and more powerful muscular sucker in each clamp than *D. merlangi*.

**Table 1:** Comparative metrical data for *Diclidophora merlangi* and their congeneric species. Values within the parentheses are related to the present study

**Species**	**BL** **(mm)**	**BMW (mm)**	**ARL (mm)**	**LCW (mm)**	**GL(mm)**	**Post-GL (mm)**	**Pre-GL (mm)**	**PL (mm)**	**BSD (mm)**	**No. of testes**
***D. merlang*** ***i*** 1	2.24	1.18	0.96	0.14	0.53	0.22	1.16	0.15	-	223
***D. merlang*** ***i*** 2	3.30	1.06	1.58	0.17	0.78	0.64	1.89	0.08	-	256
***D. merl*** ***ang*** ***i*** 3	9.07±‎1.98 (4.25-13.10)	3.69±0.77 (2.12-5.43)	3.61±‎0.98 (1.37-5.25)	0.28±0.40 (0.18-0.36)	1.96±0.43 (1.00-2.83)	1.34±‎0.39 (0.58-2.23)	4.93±1.20 (2.68-7.37)	0.27±0.04 (0.15-0.39)	-	201±31 (167-290)
***D. *** ***luscae*** 4	5.44-5.51	2.31-2.40	0.61-0.73	0.19-0.23	1.24-1.31	1.09-1.54	3.23-3.73	0.10-0.12	-	-
***D. merlang*** ***i*** 5	6.00±‎2.00 (4.00-10.00)	1.25±‎0.23 (0.45-1.39)	1.37±‎0.01 (0.98-1.86)	0.39±0.01 (0.28-0.47)	0.60±0.01 (0.40-0.80)	2.60±0.20 (1.83-2.80)	2.25±‎0.03 (1.25-2.62)	0.12±0.00 (0.08-0.15)	0.12±0.02 (0.08-0.15)	0.22±‎0.40 (0.19-0.25)

BL: Body length, BMW: Body maximum width; ARL: Anterior region length; LCW: Larger clamp width; GL: Germarium length; PL: Pharynx length; and BSD: Buccal sucker diameter.

1 : *Gadus morhua* - North Sea,

2 : *Gadus morhua* - Celtic Sea,

3 *Merlangius merlangus* - Celtic Deep,

4 : *Trisopterus luscus* and

5 : *Saurida undosquamis* - Red Sea, Egypt.

Furthermore, specimens of *D. paracoelorhynchi* are up to twice as large as *D. merlangi*, have a lobed seminal receptacle and 40-60 testes. The morphological traits of the forms isolated from lizardfish conform to those of *D. merlangi* isolated previously either from the cod *Gadus morhua* ^16^ or *Merlangius merlangus*.

**Table 2:** Comparative metrical data for *Loxuroides pricei* and their congeneric species. Values within the parentheses are related to the present study

**Species**	**BL (mm)**	**MBW(mm)**	**BWO(µm)**	**BSD (** **µ** **m)**	**EL (µm)**	**PL (µm)**	**HW ** **(mm)**	**Spines** *	**Spines** ^†^	**Testes**	**Clamps**
***L. sasikala*** 1	4.30-6.00	4.30-6.00	-	52-60×40-45	-	40-45×28-37	1.50-1.80	64-68	50-60	70-80	78-91
***L. fungilliformis *** 2	1.39-2.23	1.39-2.23	159-297	11-29×15-21	-	16-29×13-29	0.54-0.82	44-59	8-12	8-13	26-38
***L. pricei*** 3	2.66-7.27 (5.08)	2.66-7.27 (5.08)	322-684 (486)	45-74×33-45	248-374 (281)	37-53×29-41	0.98-2.46 (1.929)	72 (63-79)	34 (22-46)	56 (52-65)	47-118 (94)
***L. pricei*** 4	1.49±0.50 (1.33-5.25)	1.49±0.50 (1.33-5.25)	350±15 (220-430)	58±5×42±7 (50-65×39-47)	250±19 (225-350)	38±2×56±4 (30-45×50-65)	1.41±0.02 (0.92-2.23)	40 (35-45)	35 (25-45)	60 (55-68)	65 (60-70)

BL: Body length, MBW: Maximum body width; BWO: Body width at level of ovary; EL: Esophagus length; BSD: Buccal sucker diameter; and HW: Haptor width.

*† represent number of spines in genital atrium and cirrus, respectively. Two pairs of anchor were detected in all four species.

1 : *Cypselurus oligolepis *- India,

2 : *Hemirhamphus guoyi* - China,

3 : *Cypselurus naresii* - Gulf of Tonkin off Vietnam, and

4 : *Pagrus pagrus - *Red Sea, Egypt.

The copulatory organ of both species consisted of muscular penis with crown of 15-19 grooved and recurved hooks. The testes were noticeably smaller in the monogenean described herein; the number of testes of *D. merlangi* from the lizardfish was within the range of *D. merlangi* from the other species. Apparently, it is not the first time that *D. merlangi* has been observed on an unusual host, ^45^ since, it has been proposed that specimens of *Diclidophora gadi* (Reichenbach-Klinke, 1351) currently considered invalid^19^ on haddock *Melanogrammus aegleﬁnus*,^2^ actually represented misshapen specimens of *D. merlangi*. 

Family Axinidae^11^

Subfamily Axinoidinae^25^

Genus Loxuroides^25^

*Loxuroides pricei* ^46^

**Fig. 3:**
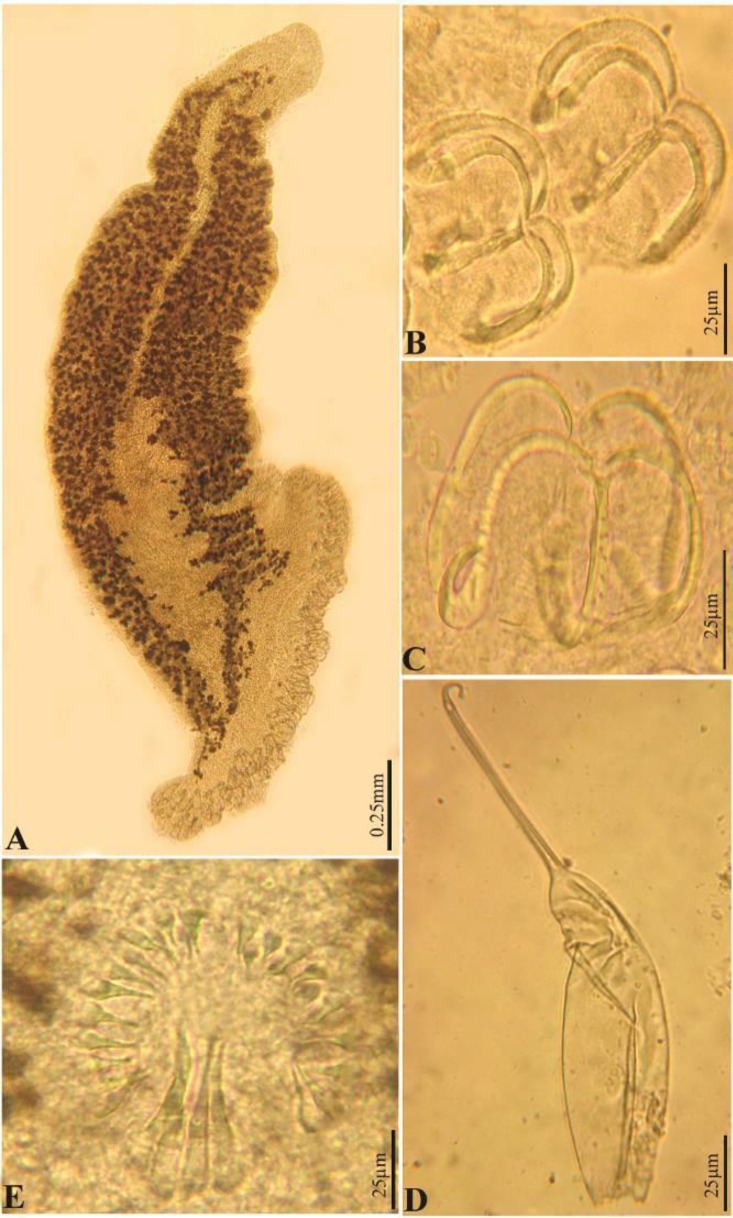
Photomicrographs of *Loxuroides pricei. *(A) Whole mount; (B) and (C) Clamps; (D) Egg; (E) Spines in genital atrium and cirrus.

**Fig. 4:**
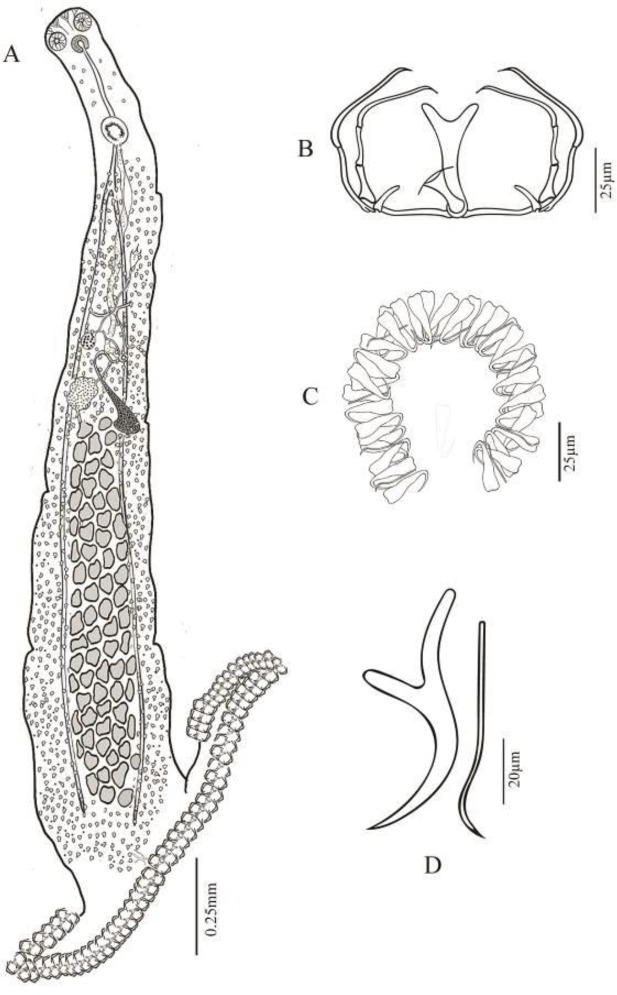
*Loxuroides pricei *from seabream *Pagrus pagrus. *(A) Total view; (B) Clamp; (C) Haptoral axinid anchors; (D) Spines in genital atrium and cirrus

The morphometric and anatomical characteristics of *Loxuroides pricei* are presented in Table 2 and Figures 3 and 4. 

**Diagnosis.** Body was slender, elongated, tapered anteriorly, posterior extremity truncated. It was 1.49 ± 0.50 (1.33-5.25) mm long and 0.51 ±‎ 10.00 (0.36-0.75) mm wide at the testicular region. Mouth was oval and subterminal, provided with two spherical buccal suckers which were aseptate, muscular and posteriolateral to mouth, measured 58.00 ± 5.00 × 42.00 ± 7.00 (50.00 - 65.00 × 39.00 - 47.00) µm in diameter. Prepharynx was short and pharynx was oval with a diameter of 38.00 ± 2.00 × 56.00 ± 4.00 (30.00 to 45.00 × 50.00 to 65.00) µm. Esophagus was slender, bifurcated just posterior to genital atrium. Two intestinal caeca extended posteriorly into haptor region with a length nearly equal. Sixty (55-68) testes, smooth, irregular spherical to oval or rectangular, extended from the posterior region of the ovary to the haptor area. Vas deferens slightly winded forward, bent at vaginal pore and running straight to the base of cirrus. Cirrus was cushion-shaped, muscular, armed with spines and formed closely set conical group, 35 (25 to 45) in number. Genital atrium was horseshoe-shaped, muscular rim armed with recurved hooks in double incomplete circle rows along inner margin. Ovary was almost equatorial, J-shaped, just pre-testicular. Ootype was elliptical, surrounded by Mehlis’ cells, located in anterior to ovarian region. Seminal receptacle was ovoid, at midway of vaginal canal. Vaginal aperture was dorsolateral, irregularly oval, armed with horn-like spine. Vitellarium follicular was predominantly extra-intestinal; follicles in two lateral non-confluent fields extended from just posterior to intestinal bifurcation to distal end of caeca but not entered haptoral region.

Vitelline reservoir was T-shaped; median vitelline duct extended posteriorly parallel to uterus, joined ootype. Uterus arose from the anterior margin of ootype, extended straight forward and opened at the unarmed uterine aperture. Eggs were oval, 250.00 ± 8.00 (205.00 to 270.00) µm long, with 103 (90-120) long filaments at anterior pole.

 Taxonomic summary

*Type host:* The common seabream, *Pagrus pagrus *(Family: Sparidae).

*Type locality:* Hurghada coasts along the Red Sea, Egypt.

*Infection site:* Gill filaments.

*Prevalence: *15 out of 35 (42.90%) samples of the examined fish were naturally infected.

*Specimens deposited: *Permanent slides were kept in Zoology Department Museum, Faculty of Science, Cairo University, Cairo, Egypt.

*Etymology: *The species is named in honor of Professor Emmett W. Price for his great contribution to the classification of the Axinidae.

**Remarks. **The genus *Loxura* was established with the type species *Loxura sasikala* from the Indian fish *Cypselurus oligolepis*.^20^ It was removed from *Loxura* and the type species *Loxuroides *arose previously.^25^ The type and the only species of this genus were *L*. *sasikala;* ^20^ *L. pricei*^46^ from *Cypselurusnaresii* in the Gulf of Tonkin in Vietnam and *L. fungilliformis *^47^ in China. According to the presence of an armed genital atrium with incomplete rows of spines and muscular cushion-shaped cirrus arming with conical spines, the present described species should belong to the genus *Loxuroides*. In comparison with the other members of the genus, it is morphologically more similar to *L. pricei* than *L*. *sasikala* and *L. fungilliformis *(Table 1). It resembles *L. pricei* in most of the body dimensions and the number of spines in the genital atrium (60-75 vs 63-79) as well as the number of spines on the cirrus. It was differentiated from *L*. *sasikala* by the shorter distance from the anterior extremity to the genital atrium, fewer testes (52-68 vs 70–80) and the number of spines in the genital atrium. Also, it is differed from *L. fungilliformis* by the long body and more number of clamps (50-68 vs 26-38), testes (52-68 vs 8-13) and spines on cirrus (25-45 vs 8-12) and genital atrium (60-75 vs 44-59). In addition, the host fish of *L*. *sasikala* and *L. pricei* are flying fishes of the family (Exocoetidae) and of *L. fungilliformis* is the half beak fishes of family (Hemiramphidae), while the present parasite was isolated from seabream *Pagrus pagrus* of the family (Sparidae), so it is considered as new host and locality records in Egypt.
